# The 5-year overall survival of cervical cancer in stage IIIC-r was little different to stage I and II: a retrospective analysis from a single center

**DOI:** 10.1186/s12885-021-07890-w

**Published:** 2021-02-27

**Authors:** E Yang, Shuying Huang, Xuting Ran, Yue Huang, Zhengyu Li

**Affiliations:** 1 Department of Gynecology and Obstetrics, West China Second University Hospital, Sichuan University, Chengdu, 610041 People’s Republic of China; 2 Key Laboratory of Birth Defects and Related Diseases of Women and Children (Sichuan University), Ministry of Education, Chengdu, 610041 People’s Republic of China

**Keywords:** Cervical cancer, The revised 2018 International Federation of Gynecology and Obstetrics (FIGO) staging guideline, Overall survival, Lymph nodes metastases

## Abstract

**Background:**

The 2018 International Federation of Gynecology and Obstetrics (FIGO) staging guideline for cervical cancer includes stage IIIC recognized by preoperative radiology (IIIC-r) to state there are lymph nodes metastases (LNM) identified by imaging tools. We aim to explore the reasonability and limitations of stage IIIC-r and try to explore the potential reasons.

**Methods:**

Electronic medical records were used to identify patients with cervical cancer. According to the new staging guidelines, patients were reclassified and assigned into five cohorts: stage I, stage II, stage IIIC-r, LNM confirmed by pathology (IIIC-p) and LNM detected by radiology and confirmed by pathology (IIIC r + p). Five-year overall survivals were estimated for each cohort. The diagnosis accuracy of computed tomography (CT), magnetic resonance imaging (MRI) and diameter of detected lymph nodes were also evaluated.

**Results:**

A total of 619 patients were identified. The mean follow-up months were 65 months (95% CI 64.43–65.77) for all patients. By comparison, the 5-year overall survival rates were not statistically different (*p* = 0.21) among stage IIIC-r, stage I and stage II. While, the rates were both statistical different (*p*<0.001) among stage IIIC-p, IIIC r + p and stage I and stage II. The sensitivities of CT and MRI in detecting LNM preoperatively were 51.2 and 48.8%. The mean maximum diameter of pelvic lymph nodes detected by CT cohort was 1.2 cm in IIIC-r cohort, and was 1.3 cm in IIIC r + p cohort. While, the mean maximum diameter of pelvic lymph nodes detected by MRI was 1.2 cm in IIIC-r cohort, and was 1.48 cm in IIIC r + p cohort. When the diagnosis efficacy of the diameter of pelvic lymph nodes in detecting LNM were evaluated, the area under the receiver operating characteristic curve (ROC curve) was 0.58 (*p* = 0.05).

**Conclusions:**

It seems that the FIGO 2018 staging guideline for cervical cancer is likely to has certain limitations for the classification of those with LNM. CT or MRI, however, has limitations on detecting LNM. It would be better to use more accurate imaging tools to identify LNM in the clinical practices.

## Background

There were 570,000 cases and 311,000 deaths of cervical cancer in 2018 globally. In Global Cancer Statistics 2018, cervical cancer ranks as the fourth most-common cancer worldwide and the second in incidence and mortality behind breast cancer in developing countries [1]. The incidence of cervical cancer is decreasing in the developed countries but is still increasing in developing countries [1].

Cervical cancer has been staged clinically [2]. The most widely used staging guidelines for cervical cancer is the International Federation of Gynecology and Obstetrics (FIGO) staging guidelines. Before 2018, gynecologists staged patients mainly by physical examinations and imaging was the auxiliary [3]. Different from some types of cancer, the prognosis of cervical cancer seems not highly effective. Increasing studies have emerged and implied that lymph node status was a key prognostic factor [4–6]. Therefore, the 2018 revised FIGO cervical cancer staging guideline includes the use of imaging for staging and allows pathological results to modify the staging [7]. In this staging guideline, patients with the involvement of pelvic and/or para-aortic lymph nodes, irrespective of the tumor size and extent are staged as IIIC, with r and p notations (r represents imaging indicating the nodes metastasis and p is pathology confirming the metastasis). Patients with pelvic lymph node metastasis are only staged as IIIC1, and those with para-aortic nodes metastasis are staged as IIIC2.

A good staging guideline is able to define the extent of the cancer and differentiate survival outcomes [8]. However, there are some controversial comments to the revised 2018 FIGO staging guideline. Some studies pointed out that the survival rates were not consistent with the stages. For example, patients in stage IIIC1 had a higher survival rate than that of in stage IIIA and IIIB [9].

In corrigendum of the new FIGO staging guideline micrometastases of lymph nodes was included in stage IIIC [10]. It suggested that in the future clinical practice, more accurate approaches like ultrastaging or sentinel lymph nodes biopsy (SLNB) should be used to make it clear whether the lymph nodes exist micrometastases or not. But we did not perform ultrastaging or SLNB before so the lymph node micrometastases were not included in this study. The primary objective of this study was to explore the validity of stage IIIC recognized by preoperative radiology (stage IIIC-r). Specifically, the cervical cancer patients based on the new staging guideline is to be restaged and it is tried to determine whether the new stage IIIC-r was able to improved 5-year survival rate differentiation with potential reasons.

## Methods

Data of patients with cervical cancer, confirmed by histology, were identified from Electronic Medical Record System of West China Second University Hospital (Chengdu, P.R. China) from January to December 31 in 2016. Written informed consents were obtained from all those patients, and the Institutional Review Board of West China Second University Hospital approved the study.

In the hospital’s medical record system, the complete medical records for every outpatients and inpatients like patient socio-demographics, tumor characteristics, first course of treatment before disease progression or recurrence, follow-up and partial survival can be found. For the ones with incomplete follow-up, we then investigated through questionnaires or called the patients or their family and got the detailed information for their disease progression, recurrence, survival or death. For the dead patients, we asked the reasons of death, we included the patients who died for cervical cancer and excluded the patients who died for other reasons.

The inclusion criteria were patients underwent surgery firstly, patients with demographic data, complete preoperative imaging results and clinical and pathological data, patients with regular follow-up data, patients with survival data, patients died for cervical cancer (progression or recurrence or distant metastasis). The exclusion criteria were patients without complete clinical and pathological data, patients underwent adjuvant chemotherapy or concurrent radiotherapy and chemotherapy, patients missed follow-up, patients died for other reasons.

Patients’ demographic data included age at diagnosis, and menstruation (menopause and pre-menopause). Imaging data included pelvic lymph nodes size, description and imaging methods (CT, MRI or PET-CT). Clinical and pathological data included stage of carcinoma (FIGO 2009), histological subtypes (squamous carcinoma, adenocarcinoma, adenosquamous carcinoma and others), degree of differentiation (poor, moderate and high), degree of stromal invasion (< 1/2 or ≥ 1/2), parametrial invasion (positive or negative), lymph node metastasis (positive or negative, described in the pathological reports) and lymph-vascular space invasion (positive or negative). The prognostic outcome assessed was overall survival. In order to ensure data authenticity and reliability, two investigators worked together: one collected and the other checked.

In FIGO 2018 cervical cancer staging guidelines, patients with positive lymph nodes, determined either pathologically or clinically, are classified as stage IIIC, irrespective of the tumor size and extent. If imaging indicates LNM, the stage allocation would be stage IIIC r, and if confirmed by pathological findings, it would be stage IIIC p. Among patients with stage IIIC, patients with positive pelvic lymph nodes are grouped as stage IIIC1 and women with positive para-aortic lymph nodes were as stage IIIC2.

In our past medical records for cervical cancer patients, FIGO 2009 was the guideline and the tumor size was categorized as either more than 4 cm or less than 4 cm. As a result, we lack partial data for the detailed tumor sizes. In order to guarantee the accuracy of our records and results, we did not reclassify all patients. The aim of this study was to figure out the prognostic performance of the stage IIIC recognized by preoperative radiology (IIIC-r). Therefore, we only reclassified the patients with positive LNs. All the included patients had standard surgical procedures according to the NCCN and 2009 FIGO guidelines for cervical cancer: radical hysterectomy and pelvic lymphadenectomy. Therefore, the patients with LNM identified by radiology were all confirmed by postoperatively pathology. Then we divided patients with LNM into three cohorts: IIIC-r cohort (LNM detected by preoperative radiology), IIIC-p cohort (LNM confirmed by postoperative pathology) and IIIC r + p cohort (LNM detected by both radiology and pathology). The stage of left patients remained the same as FIGO 2009 staging guidelines. Based on previous studies [11, 12], pelvic lymph nodes with diameter over 1 cm detected by CT or MRI of initial imaging data were classified as lymph nodes positive.

### Statistical analysis

Clinical and pathological characteristics were presented descriptively. Student’s t-test or nonparametric test for quantitative variables. The survival time was estimated from the date of diagnosis until death or last follow-up. Overall survival was estimated by Kaplan-Meier method and the log-rank test was used to compare the difference among groups. Sensitivity, specificity, positive predictive value (PPV), and negative predictive value (NPV) were used to describe the diagnosis accuracy of CT and MRI in detecting lymph nodes positive. ROC curve was used to describe the diagnosis value of the maximum diameter of lymph nodes in indicating lymph nodes positivity. Data were analyzed by SPSS (version 25, IBM Corp.) and GraphPad Prism 7 (GraphPad Software, Inc.). *P* values less than 0.05 were considered statistically significant.

## Results

**Fig. 1:**
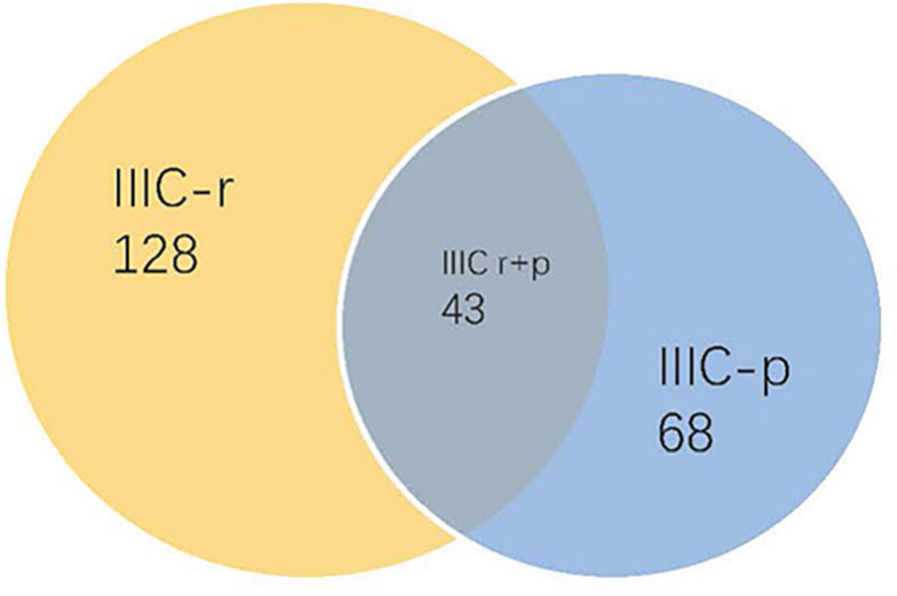
The populations of included cases in IIIC-r, IIIC-p and IIIC r + p cohorts

A total of 619 patients with cervical cancer and met the inclusion criteria were identified. The mean age of the cohort was 45 years. 74.2% (459 in 619) patients were premenopausal. The most common histological type was squamous cell carcinoma which accounted for 80.5% in all included patients. All included patients were undergone radical hysterectomy plus bilateral pelvic lymphadenectomy and confirmed by pathology. According to the preoperative imaging and postoperative pathology, the stage of 239 patients with LNM were changed from stage I or II in FIGO 2009 staging guidelines to stage IIIC-r, IIIC-p and IIIC r + p in FIGO 2018 staging guidelines. Since the patients with positive para-aortic lymph nodes were rare, patients in stage IIIC were included pelvic lymph nodes and para-aortic lymph nodes clinically or pathologically positive. Among them, 128 patients classified as stage IIIC-r cohort for the pelvic lymph nodes were positive detected by CT or MRI preoperatively only. Sixry-eight patients as stage IIIC-p cohort for their pelvic lymph nodes were positive confirmed by postoperative pathology only. Forty-three patients classified into IIIC r + p cohort for their pelvic lymph nodes were both positive in preoperative radiology and postoperative pathology (Fig. 1). The stage of the left 380 patients remained the same as FIGO 2009 guidelines. The mean age of patients was 44 years for IA-IB stage cohort, 48 years for IIA-IIB stage cohort, 46 years for stage IIIC-p cohort, 46 years for stage IIIC-r cohort and 44 years for IIIC r + p cohort. More than half patients were pre-menopausal and the most-common histologic subtype was squamous cell carcinomas in all groups. More detailed information displayed in Table 1.

**Table 1:** Clinical and pathological characteristics of all included patients in our study

Characteristics		Overall (*n* = 619)	IA-IB (*n* = 252)	IIA-IIB (*n* = 128)	IIIC-r (n = 128)	IIIC-p (*n* = 68)	IIIC-r + p (*n* = 43)
Age, y		45.85 ± 9.07	44.69 ± 9.42	48.16 ± 8.68	46.2 ± 9.42	46.22 ± 7.24	44.21 ± 8.61
Menstruation	Menopause	160 (25.8%)	51 (25.4%)	42 (32.8%)	38 (29.7%)	21 (30.9%)	8 (18.6%)
	Pre-menopause	459 (74.2%)	201 (79.8%)	86 (67.2%)	90 (70.3%)	47 (69.1%)	35 (81.4%)
Histologic subtypes	Squamous carcinoma	498 (80.5%)	199 (79%)	108 (84.4%)	106 (82.8%)	50 (73.5%)	35 (81.4%)
	Adenocarcinoma	77 (12.4%)	37 (14.6%)	14 (10.9%)	13 (10.2%)	11 (16.2%)	2 (4.7%)
	Adenosquamous carcinoma	37 (6%)	13 (5.2%)	4 (3.1%)	9 (7%)	5 (7.4%)	6 (13.9%)
	Others	7 (1.1%)	3 (1.2%)	2 (1.6%)	0	2 (2.9%)	0
Differentiation	Poor	457 (73.8%)	172 (68.3%)	95 (74.2%)	103 (80.5%)	53 (77.94%)	34 (79%)
	Moderate	115 (18.6%)	54 (21.4%)	25 (19.5%)	18 (14%)	12 (17.65%)	6 (14%)
	High	47 (7.6%)	26 (10.3%)	8 (6.3%)	7 (5.5%)	3 (4.41%)	3 (7%)
Stromal invasion	< 1/2	270 (43.6%)	143 (56.7%)	56 (43.8%)	56 (43.8%)	10 (14.7%)	5 (11.6%)
	≥1/2	349 (56.4%)	109 (43.3%)	72 (56.3%)	72 (56.3%)	58 (85.3%)	38 (88.4%)
LVSI	Positive	208 (33.6%)	61 (24.2%)	36 (28.1%)	32 (25%)	46 (67.6%)	33 (76.7%)
	Negative	411 (66.4%)	191 (75.8%)	92 (71.9%)	98 (75%)	22 (32.4%)	10 (23.3%)
Parametrial invasion	Positive	52 (8.4%)	7 (2.8%)	10 (7.8%)	7 (5.5%)	21 (30.9%)	7 (16.3%)
	Negative	567 (91.6%)	245 (97.2%)	118 (92.2%)	121 (94.5%)	47 (69.1%)	36 (83.7%)

NOTE: Values are mean (standard difference) and number of event (%). LVSI: lymph-vascular space invasion, IIIC r: patients with pelvic lymph nodes positive recognized by radiology preoperatively only. IIIC p: patients with lymph nodes positive confirmed by pathology postoperatively only. IIIC r + p: patients with pelvic lymph nodes positive recognized by radiology preoperatively and confirmed by pathology postoperatively

A summary table was to display the follow-up time and 5-year overall survival rates (Table 2). The overall mean follow-up month was 65 months (95% CI 64.43–65.77), 66 months (95% CI 66.03–67.09) for stage IA-IB cohort, 66 months (95% CI 64.9–67.15) for stage IIA-IIB cohort, 58 months (95% CI 54.31–62.19) for stage IIIC-p cohort, 65 months (95% CI 64.37–66.08) for stage IIIC-r cohort, and 59 months (95% CI 55.57–63.27) for IIIC r + p cohort. The 5-year overall survival rate was 98.8% for stage IA-IB, 97.7% for stage IIA-IIB, 79.4% for for stage IIIC-p, 96.9% for stage IIIC-r and 76.7% for IIIC r + p cohort.

**Table 2:** The mean follow-up months and 5-year overall survival among study cohorts

Cohorts	mean (m)	95% Confidence Interval	5-year survival rate
IA-IB	66.56	66.03–67.09	98.80%
IIA-IIB	66.03	64.9–67.15	97.70%
IIIC-p	58.25	54.31–62.19	79.40%
IIIC-r	65.22	64.37–66.08	96.90%
IIIC-r + p	59.42	55.57–63.27	76.70%
overall	65.1	64.43–65.77	94.50%

Note: mean(m): mean follow-up months

**Fig. 2:**
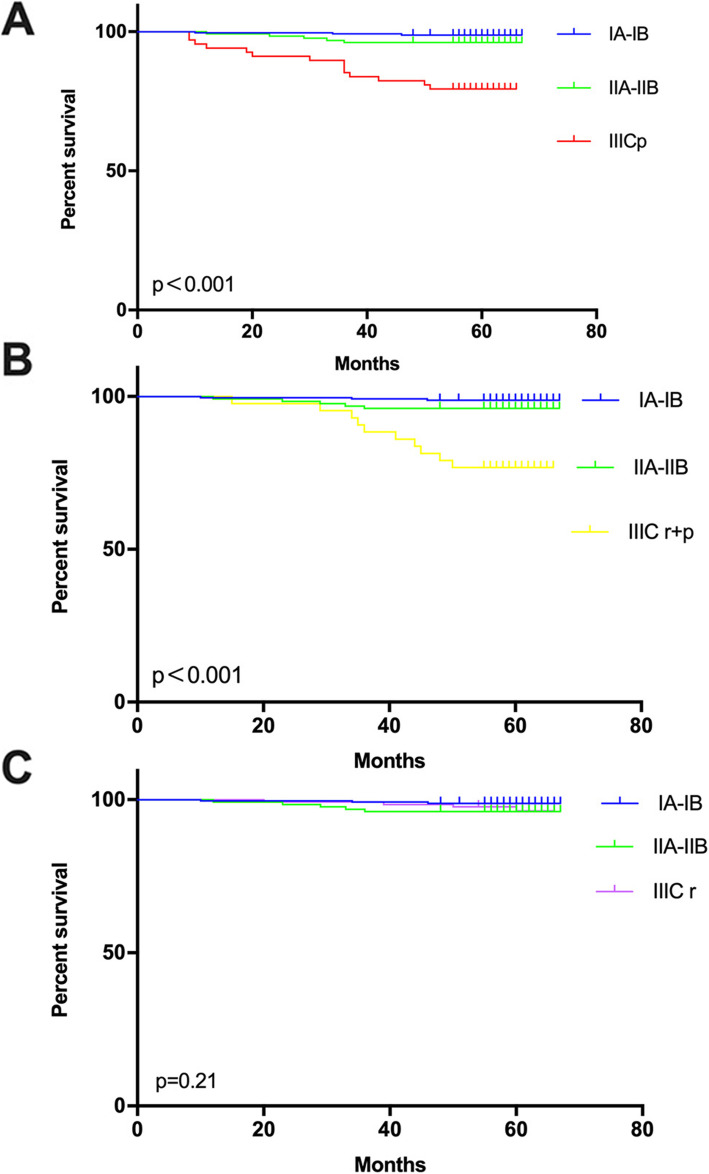
The comparisons of survival curves among different three cohorts

By comparison, the 5-year overall survival rates among stage IIIC-r cohort, IA-IB and IIA-IIB were not statistically different (*p* = 0.21) (Fig. 2c). It suggested that higher FIGO 2018 staging was less likely to be consistently associated with worse 5-year overall survival rates. When stratified based on the LNM confirmed by pathology, the 5-year overall survival rates among stage I, stage II, stage IIIC-p and IIIC r + p cohort would be all significantly and statistically different (*p*<0.001) (Fig. 2a and b). It suggested that when stratified by nodal status, there would be a decrease in survival with increasing stage.

It was then tried to explore the potential reasons for why the prognostic outcome among stage I, stage II and stage IIIC-r in this study was not consistently associated with the widely-held view: higher stage indicates a worse survival. Firstly, the diagnosis accuracy of CT or MRI in detecting adenopathy were evaluated. The sensitivities of CT and MRI were 51.2 and 48.8%, respectively. But the positive predictive values (PPV) of MRI (35.6%) was higher than that of CT (19.6%) (Table 3).

**Table 3:** The diagnosis efficacy of CT and MRI in detecting lymph nodes positive preoperatively

	CT	MRI
Sensitivity	51.20%	48.80%
Specificity	70.30%	29.70%
PPV	19.60%	35.60%
NPV	80.40%	64.40%

Note: *PPV* means positive predictive value, *NPV* means negative predictive value

**Fig. 3:**
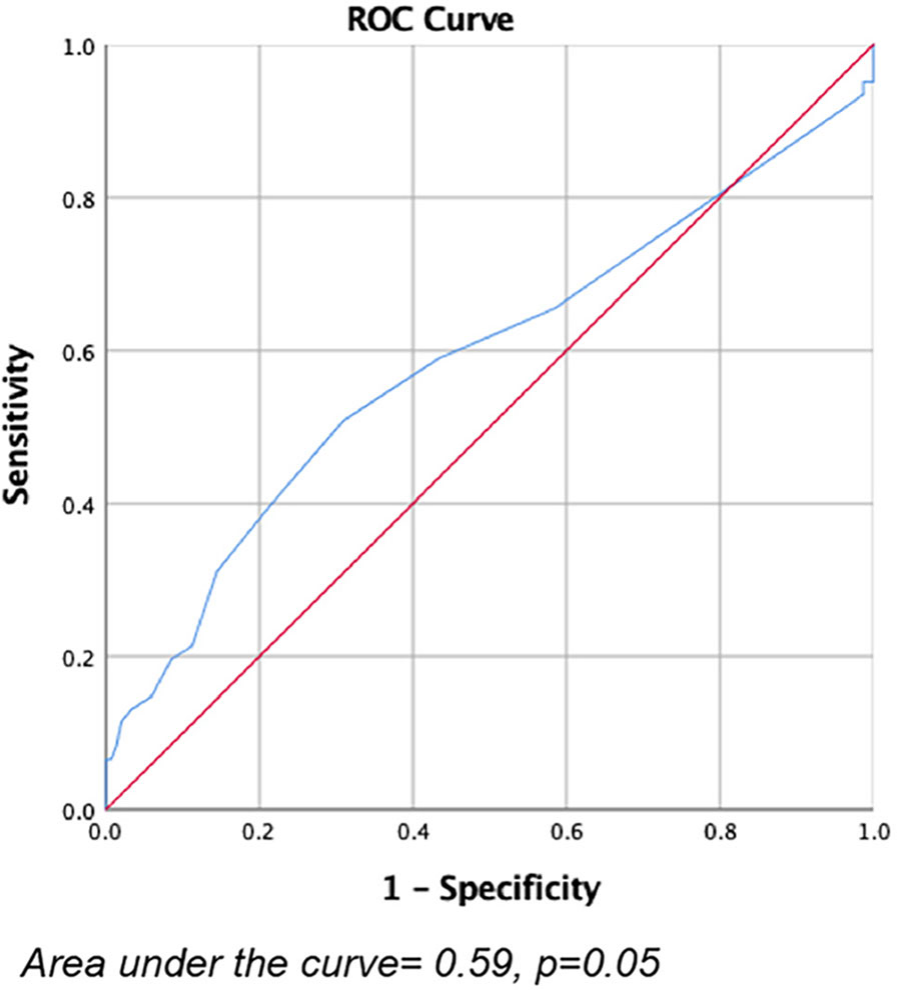
The ROC curve of the diameter of detected lymph nodes to predict the LNM

Based on previous studies [11, 12], pelvic lymph nodes with diameter over 1 cm detected by CT or MRI of initial imaging data were classified as lymph nodes positive. In this study, a total of 154 lymph nodes were detected in stage IIIC cohort. One hundred five nodes were detected by CT and 49 nodes were by MRI. Sixty-two nodes were detected in IIIC r + p cohort. Thirty-one nodes were by CT and 31 nodes were by MRI. The overall mean diameters detected by initial imaging examinations for stage IIIC-r cohort was 1.2 cm, and for IIIC r + p cohort was 1,38 cm. It indicated there was statistical difference between these two cohorts (*p* = 0.02). When using MRI, the mean diameter was 1.2 cm for stage IIIC, and 1.48 cm for IIIC r + p cohort, it indicated there was statistical difference between these cohorts (*p* = 0.02). When using CT, there seemed to be no statistical difference between these cohorts (*p* = 0.07) (Table 4). It suggested that the lymph nodes detected by initial MRI examination in IIIC r + p cohort were larger than these in stage IIIC-r cohort. When the diagnosis efficacy of the diameter of pelvic lymph nodes in detecting LNM were evaluated, the area under the ROC curve was 0.59 (*p* = 0.05) (Fig. 3). It seemed that using the diameter of lymph nodes in imaging tools to predict metastasis had little clinical significance.

**Table 4:** The maximum diameters of lymph nodes detected by CT and MRI in stage IIIC r and IIIC r + p patients

	IIIC-r		IIIC-r + p		*p* value
	n	Mean ± S.D	n	Mean ± S.D	
**Overall**	154	1.2 ± 0.26	62	1.38 ± 0.57	0.02
**CT**	105	1.2 ± 0.25	31	1.3 ± 0.33	0.07
**MRI**	49	1.2 ± 0.28	31	1.48 ± 0.73	0.02

## Discussion

Since the study was a retrospective analysis, so the data we collected were all implied the past consensus. Based on the NCCN guideline and 2009 FIGO guideline for cervical cancer, LNM was not included in the staging system and did not influenced the treatment choice. The majority patients before stage IIA could choose surgery or concurrent radiotherapy and chemotherapy and the final decision was dependent on full communication between the doctors and patients. If these patients had extensive LNM identified by preoperative imaging, the doctors would recommend the concurrent radiotherapy and chemotherapy. In our study, all included patients were underwent standard surgery and pelvic lymphadenectomy. In the follow-up, the deceased patients included in this study were died after recurrence or after distant metastases.

The data in this study implies that maybe the reasonability of stage IIIC-r in new FIGO staging system to guide prognosis is limited. After analysis, it was found that the false positive rate of LNM detected by preoperative imaging approaches like CT or MRI is high and the diagnostic accuracy for CT or MRI was relatively low. By comparison, the positive predict value of MRI was higher than that of CT. Although the diameters of metastatic lymph nodes detected by MRI was larger than those detected by CT preoperatively, the accuracy of diameter of lymph nodes to indicate metastasis was also comparatively low.

With the popularization of cervical cancer screening and vaccine, the epidemiology has been changed in many ways. Firstly, cervical cancer is controlled well in developed countries [1], but owing to the imbalance of regional development in developing countries, the incidence and mortality of cervical cancer are still high [1]. Secondly, incidence rate of early stage cervical cancer has been gradually increasing [13]. At last, the comparability of clinical stages among different countries and regions in the world has decreased, because the clinical stage in some countries, developed countries in a particular, is affected by imaging like MRI [14]. Optimal staging guidelines are more likely to keep pace with the times. Cancer tagging guidelines should be updated based on developments in diagnostic technology and reliable treatment, new results about prognostic factors and outcomes data [8]. Therefore, the revised 2018 FIGO cervical cancer staging guideline is upgraded [15].

For a long time, many researches have indicated that LNM is a poor prognostic factor [5, 16]. Yu Liu et al. indicated that the overall survival was 91% for pelvic LNM negative cohort, and 67% for nodes metastasis positive cohort [16]. LNM is the key consideration for postoperative radiotherapy. Therefore, the revised 2018 FIGO staging guideline includes the lymph nodes involvement and classified as stage IIIC, making it possible to know the lymph nodes status preoperatively detected by imaging examinations [15].

However, as showing in the results, the diagnosis accuracy of CT and MRI seem to be less ideal. In addition, the LNM of majority patients are confirmed by postoperative histology and could not be detected by preoperative imaging examinations.

It seems to be consistent with other previous studies. Based on 67 studies, Bin Liu et al. made a comprehensive comparison of CT, MRI, PET/CT and DW-MRI for detecting the LNM in patients with cervical cancer [17]. Their results showed that the sensitivity and specificity were 0.57 and 0.91 for CT detecting LNM, 0.66 and 0.97 for PET/CT, 0.54 and 0.93 for MRI, and 0.87 and 0.83 for DWI-MRI (mean ADC). By comparison, PET/CT and DWI-MRI are more likely to have a high accuracy in detecting LNM in cervical patients. As a result, the false-positive in diagnosis of stage IIIC-r is relatively high owing to the wide use of CT and MRI instead of high accurate but expensive imaging examinations like PET/CT and DWI-MRI.

The diameter of lymph nodes over 1 cm is the current diagnostic basis in most cases diagnosed as lymph node metastases by CT or MRI. In the results, the mean diameter of metastatic lymph nodes was 1.48 cm detected by MRI. However, the value of diameter indicating metastases seems to be limited. There are many metastatic lymph nodes with normal size. Meanwhile, enlarged lymph nodes could be benign lesions like inflammation or reactive hyperplasia lesions [18]. Therefore, it is hard for simple morphological characteristics to differentiate whether the lymph nodes are metastatic or not. Some studies have indicated that specific imaging features like irregular margin and central necrosis could improve the diagnosis accuracy of CT or MRI in detecting nodes metastases [19]. On the other hand, changing imaging methods could also be useful. Flurorine-18 fludeoxyglucose positron emission tomography/ CT is a useful technique in detecting the metastatic lymph nodes and could provide detailed information of the entire body [20], but owing to high cost it has not been widely used in clinical practice. Diffusion-weighted imaging (DWI) is sensitive to the diffusion of water molecules in tissue, which can make subtle abnormality more obvious and can provide better characterization of tissue and their pathological processes at microscopic level [21, 22]. With the development of technology, deep learning model could play a role on detecting adenopathy. In Qingxia Wu et al. (2020) study, they used deep learning model to identify adenopathy on magnetic resonance imaging in patients with cervical cancer. They found that the deep learning model that used both intratumoral and peritumoral regions on MRI imaging had an optimal performance, the AUC-ROC was 0.84 (96%CI 0.78–0.91) [23]. In addition, it is also important to improve the intraoperative assessment’s accuracy and the sentinel lymph node biopsy (SLNB) [24] can solve this problem. Sentinel lymph nodes (SLN) reflects the statues of the related regional lymph nodes and can be detected by lymphoscintigraphy using specific reagents like blue dye, indocyanine green [25], technetium 99 and so on, and confirmed by pathologists using frozen section, hematoxylin and eosin or ultrastaging [26] to identified micrometastases. A meta-analysis focused on SLNB in early stage cervical cancer showed that the pooled specific side sensitivity for SLNB was 88% [27].

There are some limitations in this study. Firstly, it was a retrospective analysis from a single medical center, selective bias could exist. Second, the false positive rate of stage IIIC-r was high due to the low diagnosis accuracy of CT or MRI in detecting adenopathy. Pelvic LNM plays a key role on prognosis of cervical cancer, therefore, it is important and promising to detect it preoperatively.

## Conclusion

In summary, the stage IIIC in revised FIGO 2018 staging guideline for cervical cancer is reasonable since pelvic LNM plays a key role on prognosis of cervical cancer. The routine imaging approaches like CT or MRI, however, maybe lack detailed criteria to detect LNM preoperatively. It would be better for gynecologists to use high precision imaging equipment like PET/CT or DWI-MRI to detect metastases in the future clinical practice.

## Data Availability

The datasets used and/or analysed during the current study are available from the corresponding author on reasonable request.

## Abbreviations

FIGO: International Federation of Gynecology and Obstetrics
LNM: Lymph nodes metastases
CT: Computed tomography
MRI: Magnetic resonance imaging
PET-CT: Positron emission tomography computed tomography
ROC: Receiver operating characteristic curve
PPV: Positive predictive value
NPV: Negative predictive value
SLNB: Sentinel lymph node biopsy
SLN: Sentinel lymph node
